# A Supersensitive, Multidimensional Flexible Strain Gauge Sensor Based on Ag/PDMS for Human Activities Monitoring

**DOI:** 10.1038/s41598-020-61658-z

**Published:** 2020-03-13

**Authors:** Hui Li, Jinjie Zhang, Jing Chen, Zebang Luo, Jinyong Zhang, Yousef Alhandarish, Qiuhua Liu, Wei Tang, Lei Wang

**Affiliations:** 1 Shenzhen Institutes of Advanced Technology, Chinese Academy of Sciences, Shenzhen, 518055 Guangdong China; 2 The College of Big Data and Internet, Shenzhen Technology University, Shenzhen, 518118 Guangdong China

**Keywords:** Engineering, Materials science, Nanoscience and technology

## Abstract

For more comprehensive monitoring human state of motion, it is necessary to sense multidimensional stimulus information. In this paper, we reported a supersensitive flexible sensor based on Ag/PDMS composites with sensing abilities of strain and force. The fabrication method is simple and rapid, which only need physically grinding the silver particles and mixing with liquid PDMS. The flexible sensor has excellent performances in multidimensional detection. The strain gauge factor can reach as high as 939 when it was stretched to 36%, and the minimum resolution for force detection is 0.02 N. The sensing characteristic of the sensors with different filling fraction and thickness were analyzed from the microscopic point of view. Multidimensional sensing abilities of flexible sensor have greatly expands its applications. We experimentally verified the Ag/PDMS based sensor in human body dynamic monitoring and sound detecting in real-time, which has shown great potential in motion recognition, haptic perception and soft robotics.

## Introduction

In recent years, traditional electronic devices have been expanded the range of applications by introducing wearable sensors. The characteristics of wearable sensors are flexible and comfortable, which have received extensive attention from researchers^1–6^. In particular, flexible sensors for health monitoring have becoming popular because of their enormous significant improving quality of life and changing diagnostic methods^7–11^. Flexible sensors can detect the movements of human joints and muscles^12^, external pressure on skin^13^, heartbeat^14^, pulse^15^, even respiratory frequency^16^ and some chronic diseases^17^ by directly integrating with skin or clothing. traditional wearable sensors based on metal wire, metal film^18^ or semiconductor^19^ have been widely developed and implemented. However, these sensors still lack of enough flexibility, which severely limits the application of man-machine interaction. For example, monitoring the movement of knee joint need strain sensors have stretchability over 55% strain^20^, and wearable force sensor required to have ability of skin-like elasticity to suit irregular human skin as well^21^. besides, the desired properties of flexible sensors such as good biocompatibility, lightweight, imperceptible, and inconspicuous are also indispensable^22^.

At present, with the development of material science and information technology, numerous works have been devoted to realize the above demands. Recently there have been great advances in strain sensors and force sensors. Kin Liao *et al*. prepared a CP/PDMS strain senor by high-temperature pyrolysis process. The strain sensor was highly sensitive with a gauge factor of 25.3^23^. Changyu Shen *et al*. developed a high sensitivity strain sensor (gauge factor of 79 in strain of 100%) based on reduced graphene oxide (RGO) and soft thermoplastic polyurethane (TPU) electrospun fibrous mats^24^. ChullheeCho *et al*. made a flexible tactile force sensor using conductive ink and silicon elastomer. The device had good linearity and high-selectivity measurement with a range of 0–5 N^25^. Anany Dwivedi *et al*. designed a novel haptic sensor with pyramid-shaped tactile unit and magnet embedded in a silicone rubber substrate, The sensor showed high force sensitivity, the minimum measuring force as low as 5mN^26^. Chengkuo Lee *et al*. reported various functional sensors based on the triboelectric mechanism; the wearable devices are self-powered and safe, which expand the range of application in outdoor activities^27,28^. However, those sensors could only monitor single stimulus, the collection of multidimensional of people’s information surrounding environment has been continuously required. The bioelectronics devices are not only necessary to have the flexibility similar to human skin, but also must have multidimensional sensing ability. For example, the soft glove system used in finger rehabilitation robot needs to simultaneously detect the tactile force of the fingertip and the bending angle of the knuckle, which requires the flexible sensor have both strain and force sensing capacities to monitor of applied force and tension^29–32^. Therefore, explored the sensing mechanisms involving strain and force has still significant challenging.

In this paper, we developed a high sensitive flexible sensor by uniform mixing silver particles and liquid PDMS, capable of detecting applied strain and contact force. Compared with some common techniques for fabricating flexible sensors (lithography^33,34^, screen printing^35,36^, laser cutting^37,38^, etc.), the method described here is relatively simple, low cost, and safe. The Ag/PDMS based sensors can be cut into arbitrary shapes to meet different demands. The flexible sensors have an extremely high tensile sensitivity, whose gauge factor (GF) can reach as high as 939 when it was stretched to 36%. In terms of force characteristics, our Ag/PDMS based sensor can detect force as low as 0.1 N with a minimum resolution less than 0.02 N. The sensing mechanism has been discussed by analyzing of variables internal structure of Ag/PDMS films with different filling fraction. Finally, the finger movement and sound from a phone was experimentally detecting in real-time by Ag/PDMS based sensor. The experimental results showed that the resistance change rate of the flexible sensor can reach 300% within the flexibility angle range of the finger, and the minimum sound intensity can be detected was 49.44 dB.

## Materials and Methods

### Ag/PDMS based sensor fabrication

**Figure 1:**
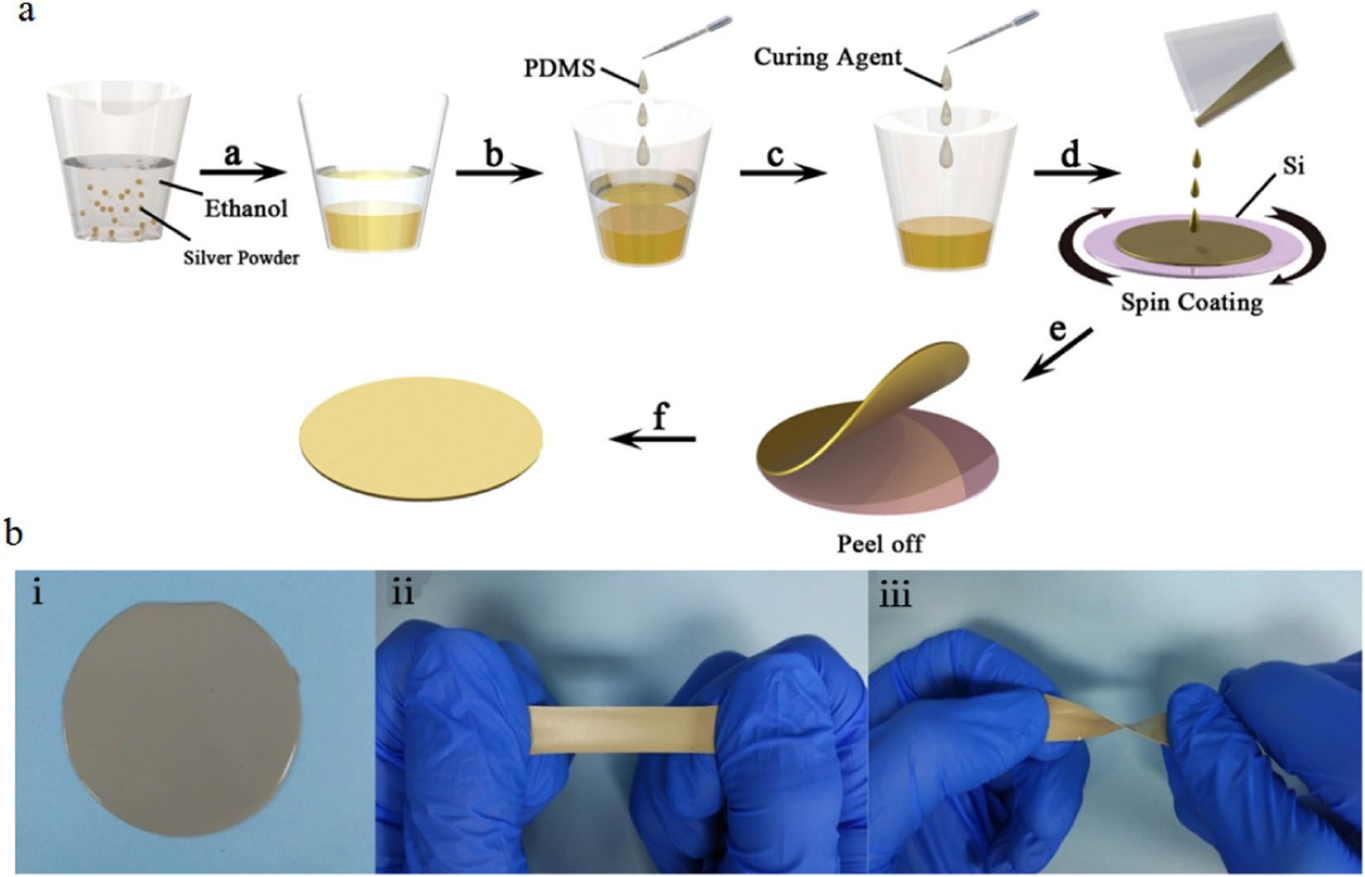
(**a**) Schematic of the Ag/PDMS based sensors fabrication procedure. (**b**) Photographs of the sensor showed excellent flexibility under stretching and twisting, respectively.

Small silver particles and PDMS were used to fabricate the flexible sensor. The fabrication procedure for the Ag/PDMS film is shown in Fig. 1a. The bulk silver powder, 99.9% (Sigma Aldrich), was preliminary refined by grinding. After added the ethanol and gently stir, the liquid mixture was ultrasonicated for 30 minutes to completely disperse the silver particles. Next, the ethanol solution on top was removed with a pipette until the silver particles settled at the bottom of plastic cup. Once the ethanol completely volatilized, PDMS (Sylgard 184) prepolymer was added to the container. The composite was mixed in a planetary mixer (Thinky ARE-250) for 3 min at 2000 rpm. A little of PDMS curing agent (a 10:1 weight ratio between the prepolymer and curing agent) drops into the Ag/PDMS mixture and further mixing by planetary mixer for 1.5 min at 2200 rpm. Next, Liquid composite poured onto a silicon wafer and spin-coating to form thin film; the thickness of film is mainly determined by the spin-coating speed and time. Finally, uncured Ag/PDMS film being hard baked at 90 °C for 15 min and gently peeled off with a tweezer from a edge of the silicon wafer. The conductive Ag/PDMS based sensor was obtained. The sensor possesses the favorable characteristics of softness and variable shape. As shown in Fig. 1b, it can be easily stretched and twisted, and quickly return to its original state after the external force released.

## Results and Discussions

The sensing capacities of resistive-type sensors are influenced by many factors, such as the filling material and amount, aspect ratio, dispersion results, and matrix material^39^. In this paper, the Ag/PDMS based sensors exhibit excellent electrical conductivity with a filler content of 150 wt.% is 1.52 × 10^−5^ Ω·m, and the change of resistance according to the filling rate of silver particles and thickness. Based on these properties, the Ag/PDMS films can be used as strain or force sensor through the encapsulation and connection of electrodes. From the repeated experiments results obtained, the conductivity threshold concentration of the Ag/PDMS composite is approximately 100 wt.%. When the filling ratio of silver powder is reach 150 wt.%, the composite has super conductivity. But composite becomes very viscous and cannot uniformly coat onto the entire silicon wafer more than 200 wt.%.

### Strain-sensing characteristic

**Figure 2:**
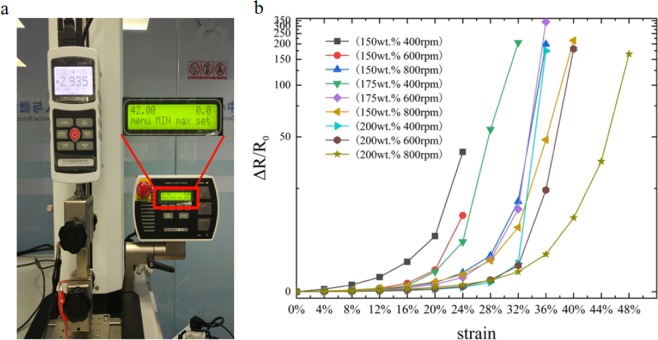
(**a**) Photographs of the Ag/PDMS based sensor was stretched more than 50% strain by the tensile testing machine. (**b**) The relationship between the strain and resistance change rate of Ag/PDMS films with different filling ratio and thickness.

**Figure 3:**
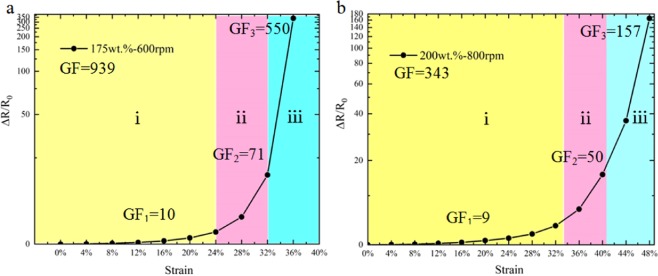
Two representative strain-resistance change rate curves (**a**: 175 wt.%-600 rpm, **b**: 200 wt.%-800 rpm) and their corresponding GF values at different stretching stages.

Here we tested the relationship between the resistance change rate and strain with various filling ratio (150 wt.%, 175 wt.%, and 200 wt.%) and thickness (400 rpm, 600 rpm, and 800 rpm). Figure 2a shows the Ag/PDMS based sensor on a tensile testing machine (MARK-10, ESM 303) subjected to more than 50% strain without failure. The results of the resistance change rate (Δ*R/R*_0_) versus strain (Δ*L/L*_0_) curves as shown in Fig. 2b. The resistance change rate of all the films increased with the raised of strain. At the initial stage, the variational tendency of each curve was gentled, and then sharply raised up when reached a certain critical point, which means that the sensitivity of the films to applied strain gradually increases. By comparing the same thickness of film with differences filling ratio, it can be concluded that, the larger filling ratio of films had smaller change in resistance at same strain, indicating that the sensitivity of the films is smaller. On the other hand, with the same filling ratio, the thicker films were, the more changed in resistance. The sensitivity of the film is higher. Sensitivity can be expressed as

1 $${\rm{GF}}=\frac{\Delta {{\rm{R}}/{\rm{R}}}_{0}}{\varepsilon }$$

where GF (gauge factor) is used to characterize the sensitivity of the stretchable sensor. The sensors can be used for specific applications according to their different sensing properties. The sensors with smaller GF values and larger strain range can be applied to large deformation measurement. Conversely, the films with larger GF values and a smaller strain suit for weak vibration detecting. We chose two representative strain-resistance change rate curves to analysis the relationship between the sensitivity and Ag/PDMS films. As shown in Fig. 3, the resistances change rates of the two curves are not linear. We divided the curves of the 175 wt.%-600 rpm film into three regions according to its GF value, each region exhibited different sensing characteristics, i.e., GF_1_ = 10 (*ε* = 0–24%), GF_2_ = 71 (*ε* = 24–32%), and GF_3_ = 550 (*ε* = 32–36%). At the region i outlined in yellow, the resistance change rate of the film is kept at a small level, and the resistance is relatively stable. Subsequently, the resistance change rate continuous increased with further stretching. The GF value is according raised much bigger than that in the region i. After reached region iii, the slope became very steep; The GF value was increased significantly and reached the maximum of 939. As shown in Fig. 3b, the variation trend curve of the 200 wt.%-800 rpm film was also similar to that of the above curve, this curve was divided into three regions as well. The GF values for each region were GF_1_ = 9 (*ε* = 0–32%), GF_2_ = 50 (*ε* = 32–40%), and GF_3_ = 157 (*ε* = 40–48%). The overall GF value of this sample could reach 343. The large value of GF indicates that the Ag/PDMS based sensor performs extremely sensitivity in strain by simple and rapid preparation method reported in this paper. The significant difference between the two strain-sensing curves is that the GF of the second curve is smaller than that of the former curve at every region. In other words, the second sample has relative lower sensitivity than the former sample during the same stretching process.

**Figure 4:**
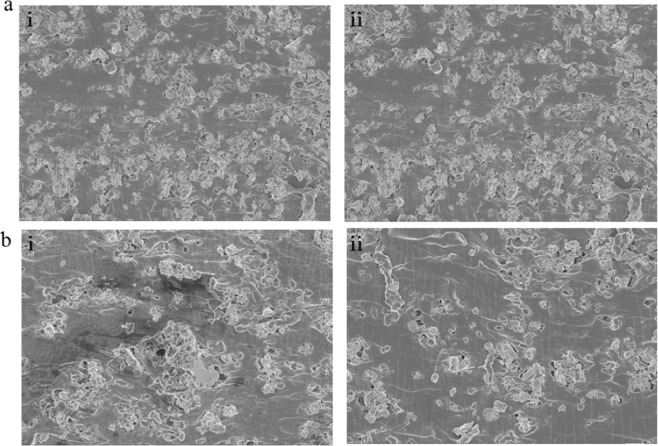
The cross-sectional SEM images of Ag/PDMS based sensors with various filling ratio: (**a**) 200 wt.%, 800 rpm and (**b**) 175wt.%, 800 rpm under states of non-deformation and 20% elongation.

We used scanning electron microscopy (SEM) to understand and explain the strain-sensing properties of the Ag/PDMS films. Figure 4 shows the distribution of silver particles before and after the film stretched (the black color shadow is Ag, the white color shadow is PDMS). When films were stretched, the distance between adjacent silver particles increased gradually along the tensile direction. Making the silver particles no longer tightly connected and the conductive pathways were reduced accordingly, which explains why the resistance of the film increases after stretching. By comparing Fig. 4a(i),b(i), the number of conductive paths in the film with 200 wt.% was deterministic greater than that in the film with the 175 wt.%. To quantitatively analyze the change of conductive paths, we also processed these SEM images using the same method. Data comparison revealed the area covered by silver particles decreased by 7.11% for the film with a filling ratio of 200 wt.% after stretching 20%. This value for the film with a filling ratio of 175 wt.% is 13.88%. Although films with different filling ratio variation rules were similar under tension, the principle of variation is different. For films with low filling ratio, the space of some silver particle become large and the conductive network is broken under applied strain, which results in an increase in the electrical resistance. However, extra loading making silver particle of high filling ratio rearrange to form new conductive paths. Therefore, the resistance change of the high filling ratio is smaller during the initial stage of stretching. This rearrangement phenomenon may rarely occur in films with a small filling ratio.

### Force-sensing characteristic

**Figure 5:**
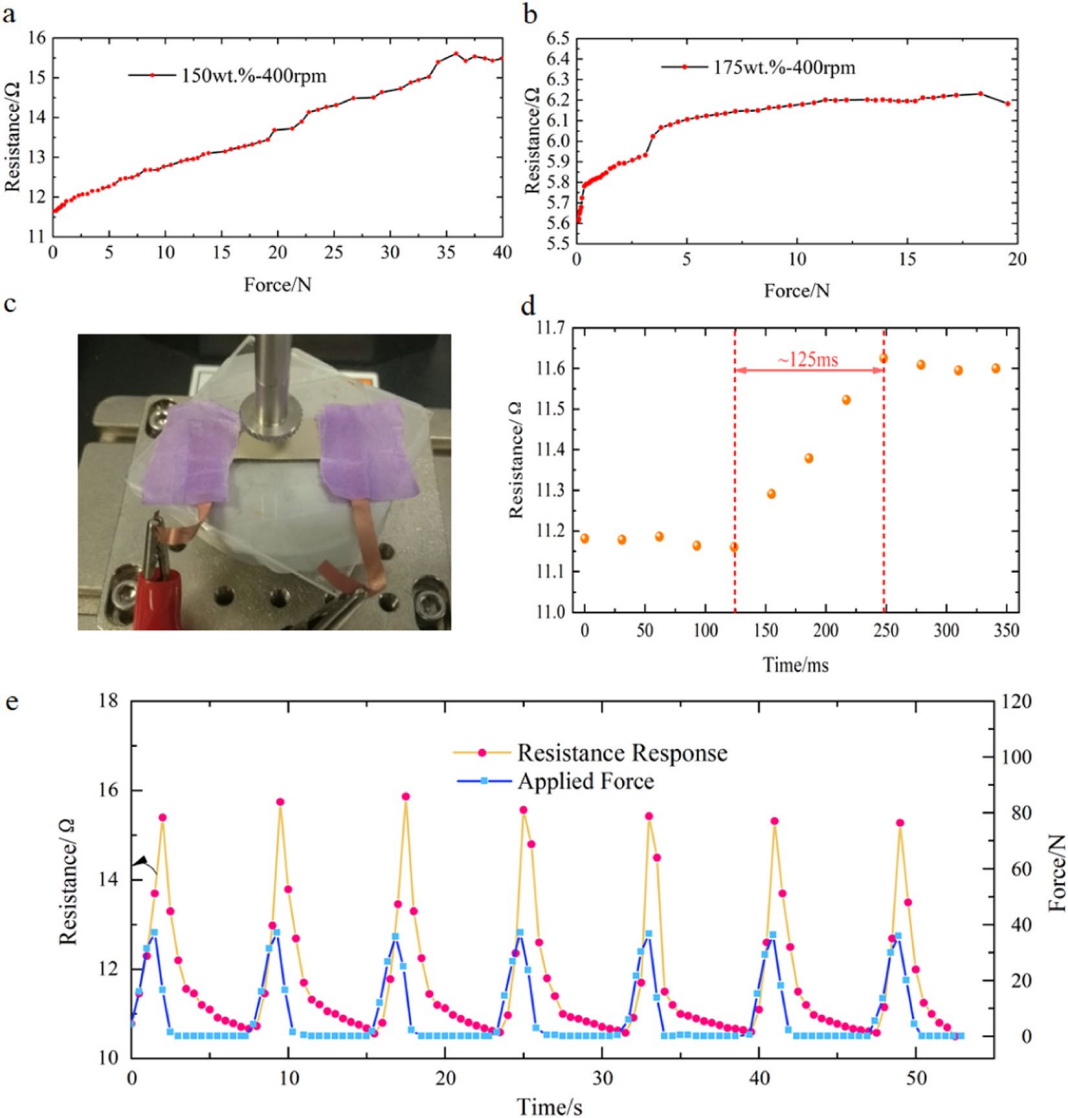
The relationship between force and resistance of Ag/PDMS films was measured with different filling ratios: (**a**) 150 wt.% and (**b**)175 wt.%. (**c**) Photograph of the Ag/PDMS film on the force testing machine. (**d**) The response time test of the film sensor with 150 wt.%. (**e**) Resistance and force change of the film under cyclic test.

Ag/PDMS based sensors also have ability of sensing force. Samples with filling ratios of 150 wt.% and 175 wt.% were selected for testing and discussion. The film deformation under pressing was much smaller than the tensile test. In the force test, the films need to be relatively thick. Here we chose the prepared films at the spin-coating speed of 400 rpm for 12 s. The force test experiment as shown in Fig. 5c. The Ag/PDMS film was smoothly attached to the workbench of the test machine to avoid unnecessary movement of the films.

As shown in Fig. 5, the resistance of the Ag/PDMS film increased with gradually applied force. It was found from the force test that as the filling ratio of the silver particles increases, the force detecting performance of the film gradually decreases, which is manifested by a decreasing in the detection range and resolution of the force. The Ag/PDMS film with 150 wt.% filling ratios performed better in force measurement. The output resistance curve was approximately linearly and whole measurement process from 0.2 N to 36 N with a linearity error 5.35%.

Besides, the film with lower filling rate showed stable force sensitivity. While the force detection range of the film with 175 wt.% filling rate is 0.08 N–18 N, which is half of that of the 150 wt.% film. It is noted that with the force increased from 0 N to18N, the resistance only changed 0.57 Ω. In addition, when force further increased to 5 N, the variation trend became weaker. We found the minimum resolution of the film with a fill ratio of 150 wt.% and 175 wt.% is 0.02 N and 0.08 N, respectively. Learning from the test method of response time in^40^, the response time of our sensor with a filling ratio of 150 wt.% was detected and summarized in Fig. 5d; and it is approximately 125 ms from a transient force of 2 N. To assess the dynamic performance of the film, we applied cyclical force from 0 to 50 N onto the flexible sensor with a fill ratio of 150 wt.% as shown in Fig. 5e. It was calculated that the repeatability error is 3.86% and the hysteresis error is 4.16% for the sensor in Fig. 5e. The changing trail of the corresponding curves of the seven periods is basically same, which proves that the film has good durability.

**Figure 6:**
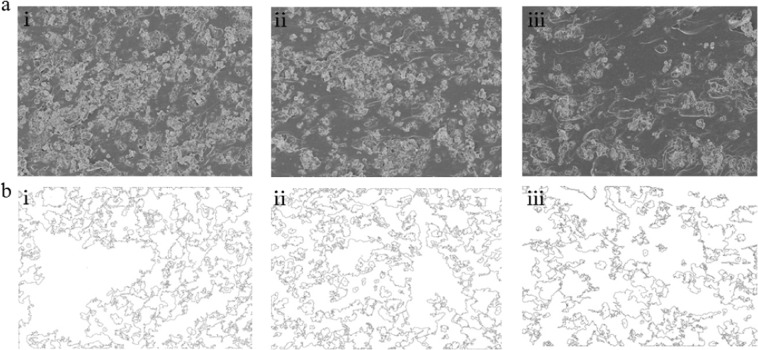
(**a**) The SEM images of cross-sections of Ag/PDMS films with filling ratio of 200 wt.%, 800 rpm; 150 wt.% 800 rpm; and 100 wt.% 800 rpm; and (**b**) their monochrome pictures.

**Figure 7:**
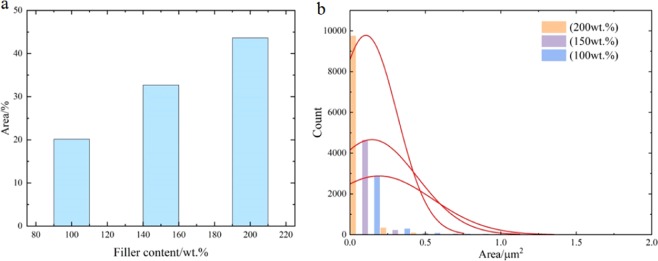
(**a**) Coverage area ratio of silver particle in the Ag/PDMS films with various filling ratio. (**b**) Aggregation area sizes distribution of tiny silver particle in the PDMS substrate with a pitch of 0.2 μm^2^.

Figure 6a shows the cross-sectional SEM images of Ag/PDMS films with various filling ratio at the original state. It can be seen from these images that the silver particle was uniformly dispersed in the PDMS substrate and became more and looser as the filling ratio decreases, indicating that the number of conductive pathways also decreases. Due to the conductivity of the Ag/PDMS films is completely determined by the composed network of silver particles, the resistance change of the films easier as the filling ratio decreases, so the silver particles distribution has significantly impacted the force sensing. We performed gray processing on the SEM images to obtain binary black-and-white images, which allows us better differentiation of silver particles from the PDMS. The result was shown in Fig. 6b, the black line is used to trace the outer contour of the silver particles. Filling ratio of silver particles and the corresponding area ratios were 100 wt.%-20.17%, 150 wt.%-32.67%, and 200 wt.%-43.64% by calculating the distribution of the black and white areas (Fig. 7a).

The area covered by silver particles increased by increasing the filling ratio, resulting in more conductive network formed inside the films. Moreover, we found numerous tiny silver particle (<3 μm^2^) were dispersed in the PDMS. As shown in Fig. 7b, the area of tiny silver particles was mainly concentrated around 0.2–0.43 μm^2^. Specifically, the smallest proportion of tiny silver particle is 93.71% of the film with a filling volume of 100 wt.%; and the largest proportion is 99.13% of the film with a filling volume of 200wt.%. The scattered tiny silver particle mainly affects the change in resistance of the film at the pressing process. The greater the ratio of silver particles filling is, the more tiny silver particles formed, making them easily recombining new conductive paths, especially in large force, resulting in a smaller variation in resistance.

### Proof of concept demonstration

The Ag/PDMS based sensor can be used as various applications duo to multidimensional sensing capacities and super sensitivity. The following experiments, we applied the flexible sensors to index finger to observe real-time finger motions and to mobile phone to sound intensity detection.

**Figure 8:**
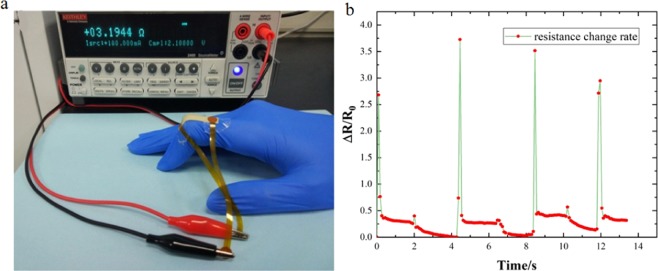
(**a**) Ag/PDMS film on index finger joint and experiment setup. (**b**) Fluctuation frequency diagram of the resistance change rate from flexible sensor deformation.

The finger movement detection experiment was performed on one of our coauthors and no participants are recruited/enrolled in the study. As shown in Fig. 8a, the Ag/PDMS film with 150wt.% was attached to the index finger by transparent dressings to detect movement of finger. The dressing only covered the electrode area, and the middle part was bare leakage, so as to avoid the influence of the dressing to the stretchability of the sensor. Figure 8b showed the resistance change rate of the sensor according to the finger state. It showed from the curve chart that the resistance change rate of the film increased to approximately 3 times at peak point with index finger bending, and the value of resistance almost returned to its initial position after relaxing the finger. The sharp responding curves indicating that the flexible sensor responded to the repeated bending-relaxing movement of an index finger really fast. This flexible Ag/PDMS sensor is possible to use for recognition of human postures and robot interface.

**Figure 9:**
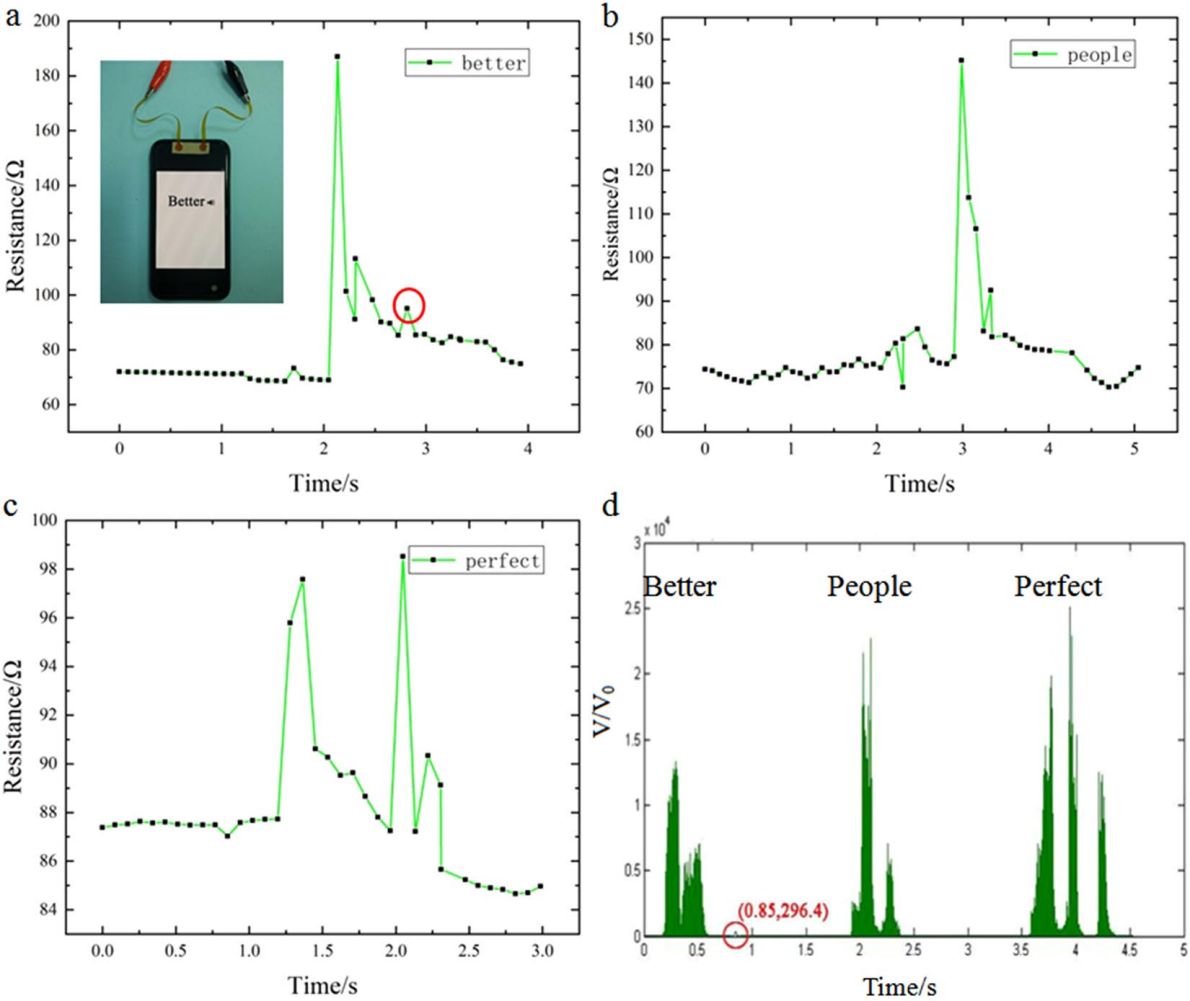
Signal responses of Ag/PDMS based sensor under different sound waves: (**a**) better, (**b**) people, and (**c**) perfect. (**d**) Audio signal waveform diagram corresponding to the three words.

We also placed this Ag/PDMS based sensor on the cell phone speaker, the attachment position of the film and the wires connection as shown in Fig. 9(a). Then, the mobile phone was played words “perfect”, “people”, and “better”. The real-time resistance changes of the film under the different sound waves as shown in Fig. 9. From these curve charts, the resistance of the film obviously increased when encountering a hard syllable; and varied relatively smaller after encountering a light syllable. To qualitatively reflect the performance of the Ag/PDMS based sensor to detect sound, the audio waveforms obtained after analyzed by Matlab software (Fig. 9d). The results showed that the position of minimum amplitude that can be detected is (0.85, 296.4), where the red circle was marked corresponding to responses (Fig. 9a). The amplitude variation of the sound can be converted to intensity of sound by the following formula.

2 $$dB=20\times {\log }_{10}(V/{V}_{0})$$

where *V*_0_ and *V* represent the standard reference value and the relative reference value of the amplitude, respectively. According to Eq. 2, we found that the minimum detection of sound intensity by the Ag/PDMS film is 49.44 dB. Based on the above characteristics, this device shows great potential as an electronic eardrum for hearing impaired individual.

## Conclusions

In this paper, we reported a multidimensional sensing flexible sensor fabricated by Ag/PDMS composites. Our method for fabricating flexible sensor is simple and rapid without corrosive or hazardous chemical reagents. The Ag/PDMS flexible sensor has good electrical conductivity (the average resistivity of the film with a filler content of 150 wt.% is only 1.52 × 10^−5^ Ω·m), high strain sensitivity (maximum GF = 939), high resolution(the minimum resolution for force detection is 0.02 N), and great stretchability (maximum strain = 50%). We experimentally verified the Ag/PDMS flexible sensor demonstrated here can be applied in a variety of applications such as human body dynamic real-time monitoring and sound intensity detecting. All of these characteristics make our sensors not only promising for motion capture, but also for softness haptic perception and human-machine interaction.
